# Asymmetric physiological response of a reef-building coral to pulsed versus continuous addition of inorganic nutrients

**DOI:** 10.1038/s41598-021-92276-y

**Published:** 2021-06-23

**Authors:** Rene M. van der Zande, Yannick R. Mulders, Dorothea Bender-Champ, Ove Hoegh-Guldberg, Sophie Dove

**Affiliations:** 1 Coral Reef Ecosystems Lab, School of Biological Sciences, The University of Queensland, St. Lucia, QLD 4072 Australia; 2 Australian Research Council Centre of Excellence for Coral Reef Studies, The University of Queensland, St. Lucia, QLD 4072 Australia; 3 Global Change Institute, The University of Queensland, St. Lucia, QLD 4072 Australia

**Keywords:** Physiology, Ecology, Ecophysiology

## Abstract

Coral reefs, especially those located near-shore, are increasingly exposed to anthropogenic, eutrophic conditions that are often chronic. Yet, corals under unperturbed conditions may frequently receive natural and usually temporary nutrient supplementation through biological sources such as fishes. We compared physiological parameters indicative of long- and short-term coral health (day and night calcification, fragment surface area, productivity, energy reserves, and tissue stoichiometry) under continuous and temporary nutrient enrichment. The symbiotic coral *Acropora intermedia* was grown for 7 weeks under continuously elevated (*press*) levels of ammonium (14 µmol L^−1^) and phosphate (10 µmol L^−1^) as separate and combined treatments, to discern the individual and interactive nutrient effects. Another treatment exposed *A. intermedia* twice-daily to an ammonium and phosphate *pulse* of the same concentrations as the *press* treatments to simulate natural biotic supplementation. *Press* exposure to elevated ammonium or phosphate produced mixed effects on physiological responses, with little interaction between the nutrients in the combined treatment. Overall, corals under *press* exposure transitioned resources away from calcification. However, exposure to nutrient *pulses* often enhanced physiological responses. Our findings indicate that while continuous nutrient enrichment may pose a threat to coral health, episodic nutrient pulses that resemble natural nutrient supplementation may significantly benefit coral health and physiology.

## Introduction

Coral reef ecosystems thrive in nutrient poor waters typical of many tropical coasts and oceans. The concentrations of inorganic nitrogen (N) and phosphorus (P) in coral reef waters are often amongst the lowest values recorded for aquatic systems^1, 2^, yet many coral reefs are typified by substantially high rates of primary productivity^3^. One mechanism enabling symbiotic corals to persist in these oligotrophic waters is the capacity of the coral holobiont to rapidly take up dissolved inorganic (e.g. ammonium and phosphate) and organic nutrients (e.g. amino acids) from the surrounding water^4–8^. Efficient nutrient recycling between the coral host and their dinoflagellate endosymbionts (Symbiodiniaceae)^9–11^ and other associated microbes^12^ further enable corals to thrive in nutrient-poor environments.

### Nutrient uptake and assimilation

Inorganic N is primarily taken up by the holobiont in the form of ammonium and nitrate^7, 13, 14^. Nitrate must first be reduced to ammonium by the symbiont using nitrate reductase before assimilation^13^. While the coral host is capable of its own ammonium assimilation through the glutamine synthetase/glutamate dehydrogenase (GS/GDH) pathway, the majority of N assimilation occurs in the symbiont through the glutamine synthetase/glutamine oxoglutarate aminotransferase (GS/GOGAT) cycle^10, 14, 15^. Inorganic P (mainly phosphate) is assimilated primarily by the symbionts^8^, though low *in hospite* symbiont phosphate content suggests that the host controls availability^16^. The exact mechanisms involved in P uptake remain largely unexplored^10, 17^, as do the relative roles of Symbiodiniaceae and coral microbial associates in P uptake and assimilation^18^.

Because symbiotic corals are so finely adapted to living under nutrient-poor conditions, increases in nutrient concentrations of the surrounding seawater due to anthropogenic sources (e.g. sewage, fertilizer runoff) often disrupt host and symbiont physiology. Under natural conditions, inorganic N and P are considered limiting factors for Symbiodiniaceae growth and photosynthesis^19–21^. Addition of these nutrients to the surrounding seawater may lead to rapid nutrient uptake by the symbiont, C:N:P ratio shifts particularly within the symbiont^22^, increased symbiont photosynthesis, and organic carbon accumulation in both the host and the symbiont^23^. Nutrient enrichment therefore likely stimulates symbiont growth at the expense of the host^24–28^. Enhanced symbiont growth consumes organic carbon faster, possibly leading to lower translocation of photosynthetic carbon to the host^29, 30^. Extreme levels of ammonium (typically ≥ 20 µmol L^−1^) may directly interfere with skeletogenesis, carbohydrate metabolism, ion transport, and host-symbiont interaction^5, 20, 31, 32^, while imbalanced N:P ratios (usually nitrate) of available nutrients may cause stress resulting in coral bleaching, photosynthetic impairment, declining biomass, and increased mortality^33–35^.

### Natural nutrient loading

Interestingly, corals naturally receive episodic pulses of nutrients several times higher than the background concentrations in the overlying waters, delivered to reefs by diurnal activities of fish and other aquatic organisms^36–38^, as well as seabirds^39, 40^. These nutrient influxes are dynamic and temporal, and usually reflect the diurnal migratory and feeding behaviour of fish^36–38^. The efficient nutrient uptake by corals could allow them to capitalize on episodic high nutrient influxes available in the waste products of organisms that live in close proximity^37, 41, 42^. Contrary to the negative effects of anthropogenic eutrophication on coral physiology, corals exposed to these temporary and natural nutrient pulses showed increased tissue thickness, CaCO_3_ accretion and skeletal expansion rates^38, 43, 44^, provided that the reef is not yet in a degraded state^5, 45^.

The conflicting outcomes of natural (and often temporary) vs anthropogenic (and often continuous) deposition of nutrients on coral reefs and the heterogeneous effects of inorganic N and P on coral physiology outline gaps in our knowledge regarding the influence of nutrients on symbiotic corals^5, 46, 47^. Firstly, it remains unclear whether elevated ammonium and phosphate are capable to interactively influence coral physiology when applied together^48^. Although an increasing number of studies address the effects of continuously elevated (*press*) levels of inorganic N and/or P on coral physiology, only few have determined their relative contribution within an orthogonal experimental design^5, 20^. Secondly, it is currently unknown how N and P affect coral functioning differently under continuous or temporary exposure^5, 48^. The present study therefore examines the effect of *press* elevated ammonium and phosphate under an orthogonal design on long- and short-term coral health (day and nighttime calcification, skeleton expansion, productivity, energy reserves and tissue stoichiometry) after a 7-week exposure in the symbiotic coral *Acropora intermedia*. Importantly, as a simulation of natural nutrient fluxes of biological origin on coral reefs, a separate treatment assessed the effects of episodic (*pulse*) ammonium and phosphate supplementation in comparison with no supplementation (control) and the *press* ammonium and phosphate treatments.

## Materials and methods

### Experimental design

This experiment was performed on Heron Island, located approximately 70 km off the eastern shore of Australia on the southern Great Barrier Reef, and unaffected by terrestrial run-off^49^. Despite significant guano deposition by the resident seabird population^39, 40^, average seawater conditions around Heron Island are mostly oligotrophic (0.49–0.55 µmol L^−1^ ammonium and 0.13–0.26 µmol L^−1^ phosphate) but can temporarily exceed 20 µmol L^−1^ ammonium and 4.5 µmol L^−1^ phosphate^40, 50^. During this study, seawater concentrations of ammonium and phosphate averaged 0.55 and 0.32 µmol L^−1^ respectively across the experimental period, with respective maxima of 0.91 and 0.62 µmol L^−1^ (Table S1, Figure S1). Seawater nitrate and nitrite concentrations were not measured during this study, but previous studies recorded concentrations comparable to ammonium^51^.

A total of 150 branch tip fragments of *A. intermedia* were collected in August 2015 on the reef flat of Heron Island between 0.5 and 1 m water depth from 15 different adult colonies. Upon returning to the lab, fragments were selected for equal thickness and trimmed to 5 cm length and suspended upright from monofilament line in 30 L aquaria with untreated flow-through seawater. Water flow was maintained at 1 L min^−1^, and all aquaria were fitted with a power head (infinity 800, Clearpond, WA, Australia) for water circulation. Fragments were randomly distributed over 20 aquaria with 7 or 8 fragments per aquarium. Aquaria were shaded to allow approx. 700 µmol quanta m^−2^ s^−1^ of PAR (LI-1400, LI-COR Inc., Lincoln, Nebraska, USA) at midday, which is comparable to light intensities measured at the coral collection site. Fragments were left to recover for 6 days, after which five separate experimental treatments (n = 4 aquaria per treatment) were initiated: (1) Control; neither ammonium nor phosphate was elevated (ambient seawater concentrations). (2) *Press* N; ammonium was permanently elevated to 14 µmol L^−1^ while phosphate was kept at ambient levels. (3) *Press* P; phosphate was permanently elevated to 10 µmol L^−1^ while ammonium was kept at ambient levels. (4) *Press* NP; ammonium and phosphate were both permanently elevated to 14 and 10 µmol L^−1^ respectively. (5) *Pulse* NP; ammonium and phosphate were elevated twice daily (09:00 and 17:00) to 14 and 10 µmol L^−1^ for 30 min each time. After 30 min excess nutrients were flushed out and concentrations of both nutrients returned to ambient levels. These concentrations of ammonium and phosphate were chosen because they simulate severe nutrient pollution^5^, comparable to maximum values recorded for Heron Island^40^.

In the *press* treatment, the nutrient perturbation was sustained with a new equilibrium being achieved and maintained, whereas in the *pulse* treatment the perturbation was temporary, after which the system returned to its original equilibrium^52^. *Press* treatment conditions were created by continuously feeding a concentrated ammonium (4.7 mmol L^−1^) and/or phosphate (3.3 mmol L^−1^) solution into the aquaria. Solutions were prepared fresh daily with 0.45 µm filtered seawater (FSW) and kept in darkened 20 L sumps. Each solution was dripped into the aquaria using small pumps (Aquagarden Mako 4000, Clearpond, WA, Australia) set at 3 ml min^−1^. Normal untreated seawater flow into the aquaria was maintained at 1 L min^−1^. Untreated seawater flow and nutrient drip speeds were monitored and adjusted daily. Nutrient pulses in the *pulse* treatment were added twice daily at 09:00 and 17:00. During the pulse, water flow and nutrient drips (but not water mixing) to all aquaria (*press* and *pulse*) was interrupted for 30 min, after which it was resumed normally. Tubing for the nutrient drips was darkened, and nutrient sumps were completely emptied and cleaned weekly to prevent algae growth. Treatment aquaria were cleaned daily, and water samples taken from the aquaria were analysed for ammonium and phosphate concentrations. Ammonium and phosphate assays were carried out using a photometric approach after ^53^ (pp. 14–17 and pp. 22–25 for ammonium and phosphate respectively). Corals were kept under treatment conditions for 7 weeks before physiological measurements were done.

### Metabolic parameters

Coral net photosynthesis (P_NET_) and dark respiration (R_DARK_) rates (n = 12 per treatment) were obtained from metabolic oxygen flux measurements over light–dark cycles. Corals were incubated in sealed 250 ml acrylic chambers under 700 µmol quanta m^−2^ s^−1^ (Aqua Medic Ocean Lights, Aqua Medic, Bissendorf, Germany). Chambers were filled with 0.45 µm FSW from the respective *press* treatments. Corals from the *pulse* NP treatment were incubated under untreated (ambient) seawater conditions to obtain a response more reflective of the overall treatment history. Starting seawater oxygen content was reduced to approximately 60% air saturation (60.8 ± 5.1, mean ± sd) by N_2_ gas bubbling, possibly also slightly affecting carbonate chemistry. Chambers were temperature controlled using a water bath (Julabo F33ME refrigerated/heating circulator, Seelbach, Germany). Oxygen concentration in the chambers was logged at 15 s intervals using a PreSens OXY-10 mini oxygen meter (PreSens, Regensburg, Germany) over 30–30 min light–dark cycles. P_NET_ and R_DARK_ rates were determined from the light measurements and after a 20 min dark adaptation period respectively to determine holobiont potential for remaining net photosynthetic over a 24-h period, and to estimate the amount of photosynthetic carbon potentially available for translocation to the host.

### Skeletogenesis

Rates of day and night calcification (G_TA_) were measured using the total alkalinity (TA) anomaly technique^54^. Corals (n = 12 per treatment) were incubated for 1 h in temperature-controlled (Julabo F33ME refrigerated/heating circulator, Seelbach, Germany) 250 ml acrylic chambers containing 0.45 µm FSW from the respective *press* treatment, or untreated (ambient) seawater for the *pulse* NP treatment. ΔTA was measured by Gran titration after ^55^, and corrected for the minimal ammonium and/or phosphate fluctuations (Table S2 and Supplementary methods) in the chambers during the incubation^56^.

### Tissue composition

After the incubations, coral specimens (n = 12 per treatment) were frozen for tissue analysis (detailed in Supplementary methods). Briefly, tissue was removed from the skeleton using an airbrush and the resulting tissue mixture was vortexed and stored at -20 °C. Water-soluble host protein concentration was determined by differential absorbance at 235 and 280 nm through spectrometry (Spectra Max 2, Molecular Devices, Sunnyvale, California) according to ^57^. Total lipid concentration was quantified from freeze-dried samples (ScanVac CoolSafe, LaboGene, Lillerød, Denmark) after extraction in chloroform/methanol (2:1) solution and subsequent washing in 0.1 mol KCl and methanol/MQ (1:1) solutions^58, 59^.

Holobiont total organic carbon (TOC) content and N:P ratios were determined from freeze-dried samples (ScanVac CoolSafe, LaboGene, Lillerød, Denmark) at the Analytics Lab at the School of Agriculture and Food Sciences, The University of Queensland. Total nitrogen (TN) was analysed by combustion analysis (LECO TruSpec analyser, Michigan, USA), total phosphorus (TP) by acid digestion and inductively coupled plasma optical emission spectrometry (ICP-OES) analysis, and TOC was analysed by combustion (LECO TruSpec analyser, Michigan, USA) on acidified samples to remove carbonates. In order to meet the minimal analysis weight, samples from each tank (n = 4) were pooled and analysed at tank level.

After removal of the tissue, the coral skeletons were treated with 10% hypochlorite solution for 12 h until completely white and all remaining tissue had been removed^60^. Skeletons were then rinsed in fresh water and dried overnight at 60 °C. End-of-experiment skeleton surface areas of the coral fragments were assessed using the double waxing method^61^. Surface areas of a fragment subset (n = 20) taken at the start of the experiment were measured to confirm that initial surface areas of the fragments used in the experiment were comparable (13.66 ± 1.53 cm^2^; mean ± sd).

### Statistical analysis

The interactive effect of ammonium and phosphate (between the control, *press* N, *press* P and *press* NP treatments) on photobiology, tissue lipid and protein content, and skeleton surface area responses were tested in a nested three-factorial ANOVA design (factors: Tank, Ammonium and Phosphate). In this design, Tank was nested in the interactive effect of categorical factors Ammonium and Phosphate (levels: ambient and elevated). Due to the pooling of the samples and analysis at tank level, holobiont N:P ratios and TOC content were analysed in a two-factorial ANOVA design (factors: Ammonium and Phosphate; levels: ambient and elevated). Day and Night rates of G_TA_ were individually explored for tank effects in a preliminary analysis, and after none were detected (Table S3) these variables were further analysed using a three-factorial repeated measures design with categorical factors Ammonium and Phosphate (levels: ambient and elevated), and Time (Day and Night) as the within subject factor. The effect of continuous vs episodic nutrient loads (*press* vs *pulse*) was tested for all variables except G_TA_ and tissue TOC content and N:P ratio, between the control, *press* NP and *pulse* NP treatments in a nested one-way ANOVA, where tank was nested in the treatments. Here, G_TA_ was analysed in a repeated measures one-way ANOVA after it was determined in a preliminary analysis that there were no tank effects (Table S3), and tissue N:P ratio as well as TOC content were analysed at tank level in a one-way ANOVA. In all *press* vs *pulse* comparisons, significant outcomes were further explored using Tukey HSD *post-hoc* analyses. All analyses were tested for violations in assumptions for normality (Shapiro–Wilk test) and homogeneity of variances (Levene’s test), and the data were log or square root transformed where necessary. Analyses were performed to the α = 0.05 significance level. Heteroscedastic datasets were assessed at α = 0.01 significance level^62^. All statistics were performed using Statistica 13.2 (Statsoft, Tulsa, OK, USA).

## Results

### Skeletogenesis

**Figure 1:**
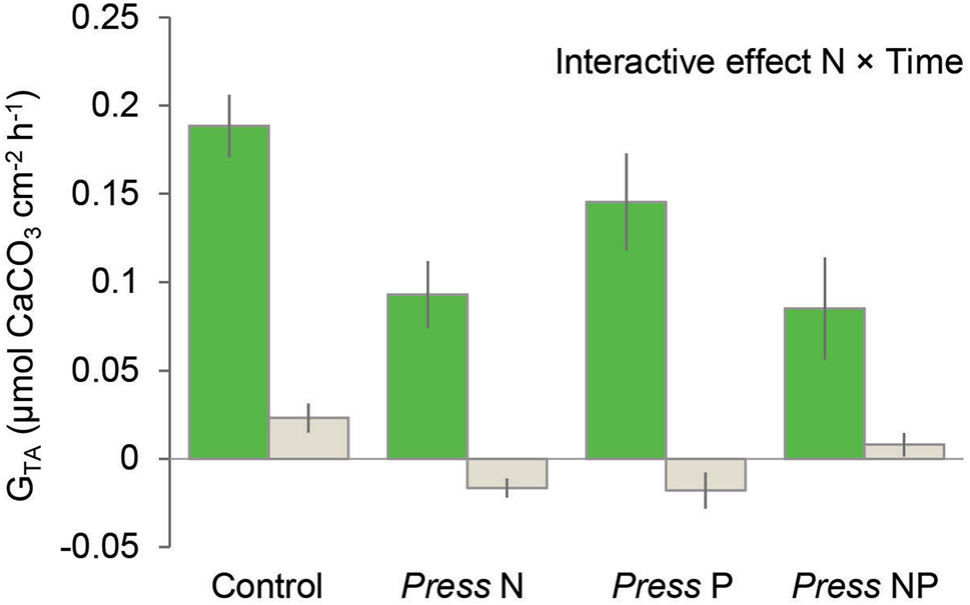
Rates of calcification measured at the end of the experiment (G_TA_, n = 12) for *Acropora intermedia* in an experimental study done on Heron Island on the southern Great Barrier Reef. Corals were exposed for 7 weeks under an orthogonal design to continuous (*press*) addition of ammonium (N) and/or phosphate (P). G_TA_ rates (mean ± SE) were measured during day (green bars) and nighttime (grey bars) in one-hour light and dark incubations respectively, and standardized to end-of-experiment tissue surface areas.

Calcification rates (G_TA_) after seven weeks of treatment conditions showed a strong dependence on time of day (Fig. 1). During nighttime, rates of G_TA_ fell to below zero values in the *press* N and *press* P treatments (− 0.017 and − 0.018 µmol cm^−2^ h^−1^ respectively). Overall, G_TA_ was governed by an interactive effect between levels of ammonium, and the time of day (interactive effect Time × N; F_1,44_ = 10.98, *p* = 0.002) regardless of the level of phosphate. Elevated levels of ammonium significantly reduced daytime G_TA_, but had no effect on nighttime G_TA_.

**Figure 2:**
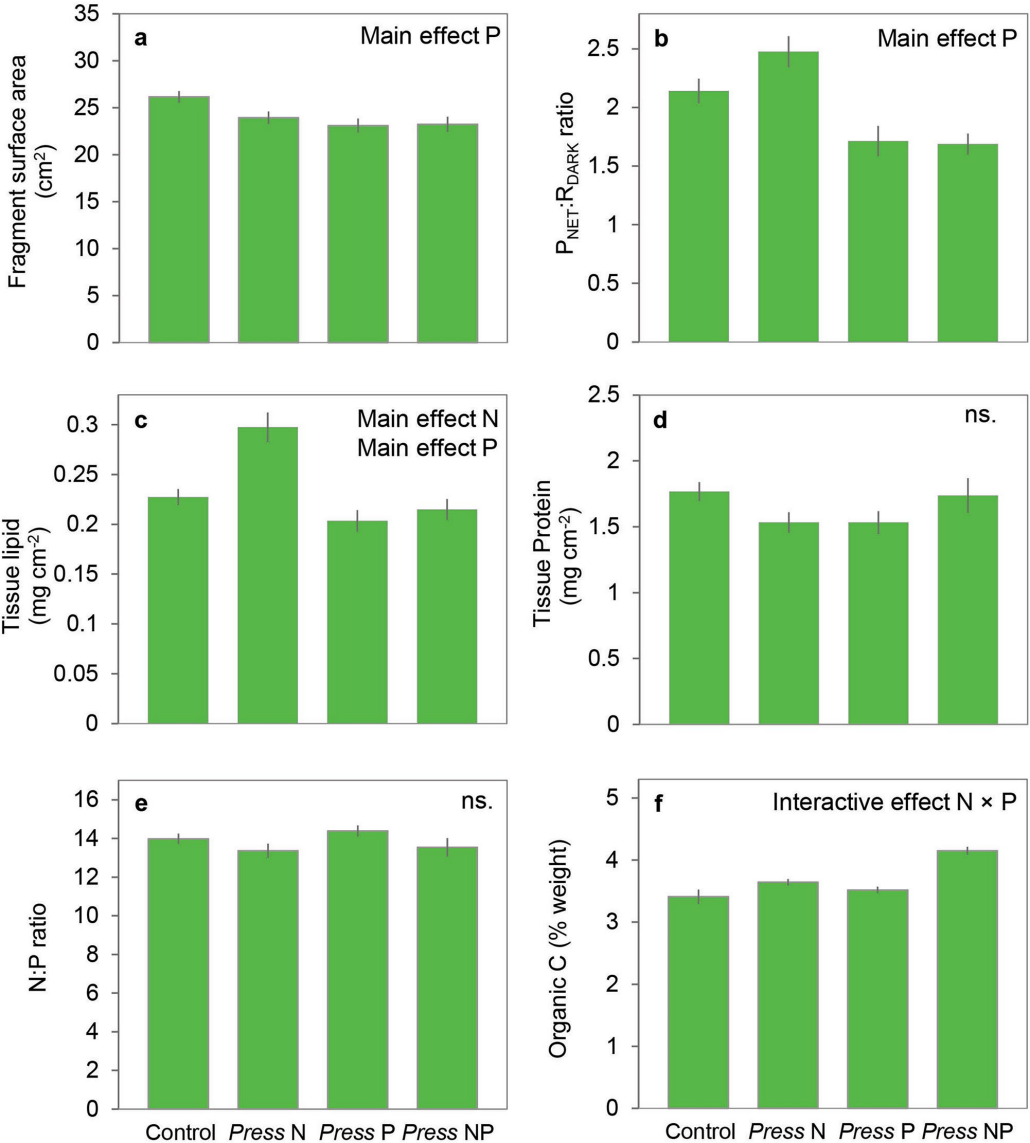
Metrics of coral physiology (mean ± SE) of *Acropora intermedia* fragments after 7-week exposure to elevated ammonium (N) and/or phosphate (P) under an orthogonal design. Fragment surface area (n = 16) was measured at the end of the experiment (**a**). Net photosynthesis (P_NET_) to dark respiration (R_DARK_) ratios (n = 12) were obtained from oxygen flux measurements (**b**), and total tissue protein (**c**) and lipid (**d**) concentrations (n = 12), and tissue N:P ratio (**e**) and organic carbon (**f**) content (n = 4) were measured from tissue samples collected at the end of the experiment.

End-of-experiment skeleton surface areas of the fragments differed significantly between treatments (Fig. 2a). Largest fragment surface areas were measured for the control treatment (26.14 ± 0.63 cm^2^; mean ± SE). Fragment surface areas were reduced when exposed to *press* elevated levels of phosphate to 23.09 ± 0.74 cm^2^ (mean ± SE), but were unaffected by levels of ammonium (main effect P; F_1,48_ = 6.66, *p* = 0.013).

### Metabolic parameters and tissue composition

Elevated phosphate levels reduced P_NET_:R_DARK_ ratios (Fig. 2b) irrespective of the level of ammonium (main effect P; F_1,32_ = 24.08, *p* < 0.001). These differences were caused by reduced P_NET_ under elevated phosphate (main effect P; F_1,32_ = 4.24, *p* = 0.048), as there was no effect of treatment on the dark respiration rate.

Host protein concentration (Fig. 2c) did not differ significantly between any of the treatments. Tissue lipid content (Fig. 2d) was influenced by exposure to ammonium (main effect N; F_1,32_ = 11.59, *p* = 0.002) and phosphate (main effect P; F_1,32_ = 19.77, *p* < 0.001), but was unaffected by their interaction. *Press* exposure to elevated ammonium increased the tissue lipid content, whereas *press* exposure to phosphate decreased tissue lipid content.

The N:P ratio of the holobiont (Fig. 2e) was not influenced by exposure to ammonium and/or phosphate, but tissue TOC content (Fig. 2f) was governed by an interactive effect of ammonium and phosphate (interactive effect N × P; F_1,12_ = 26.55, *p* < 0.001). Exposure to phosphate resulted in increased tissue TOC content under elevated levels of ammonium, but did not influence tissue TOC content under ambient ammonium levels.

### Press–pulse comparison

The results of the comparison between permanent (press), episodic (pulse) and no (control) exposure to elevated levels of ammonium and phosphate are summarized in Table 1. The rate of coral CaCO_3_ accretion (G_TA_) at the end of the experiment was significantly lower in all treatments under nighttime compared to daytime conditions (F_1,9_ = 51.06, *p* < 0.001), and also differed between treatments (F_2,9_ = 6.06, *p* = 0.022). Exposure to *press* levels of ammonium and phosphate resulted in reduced G_TA_ compared to both the control (Tukey HSD *p* = 0.031) and the *pulse* NP treatments (Tukey HSD *p* = 0.041), whereas no differences in G_TA_ were observed between the control and *pulse* NP treatments (Tukey HSD *p* = 0.982). Under *press* concentrations of ammonium and phosphate, fragment surface areas were significantly reduced compared to the control (Tukey HSD *p* = 0.046) and the *pulse* NP (Tukey HSD *p* < 0.001) treatments, while no differences in fragment surface areas were observed between the control and the *pulse* NP treatments (Tukey HSD *p* = 0.182). No significant differences in P_NET_:R_DARK_ ratios, host protein concentration and tissue N:P ratio were found between the control, *press* NP and *pulse* NP treatments. Tissue lipid content differed between the *press* and *pulse* treatments (F_2,24_ = 9.048, *p* = 0.001). Lipid concentrations were increased under the *pulse* NP treatments compared to the control (Tukey HSD *p* = 0.009) and *press* NP treatments (Tukey HSD *p* < 0.002). Pulses of ammonium and phosphate also significantly influenced tissue TOC content (F_2,9_ = 333.3, *p* < 0.001). Tissue TOC content was higher in corals in the *pulse* treatment compared to the control (Tukey HSD *p* < 0.001) and the *press* treatment (Tukey HSD *p* < 0.001). Additionally, TOC content was elevated in the *press* treatment over the control treatment (Tukey HSD *p* < 0.001).

**Table 1:** Physiological parameters (mean ± SE) measured for fragments of *Acropora intermedia* after 7-week exposure to continuously (*Press*) and temporary (*Pulse*) elevated ammonium (N) and phosphate (P) concentrations, as well as under ambient (Control) conditions.

Parameter	Treatment			Significance (nested one-way ANOVA)
	Control	*Press* NP	*Pulse* NP	
**G**_**TA**_** (µmol CaCO**_**3**_ **cm**^**−2**^ **h**^**−1**^**)** Light Dark				Light > Dark Control, Pulse > Press
	0.189 ± 0.018	0.085 ± 0.029	0.151 ± 0.022	
	0.023 ± 0.008	0.008 ± 0.007	0.054 ± 0.013	
End-of-experiment fragment surface area (cm^2^)	26.14 ± 0.634	23.23 ± 0.823	28.27 ± 0.943	Pulse > Control, Press
P_NET_:R_DARK_ ratio	2.140 ± 0.106	1.687 ± 0.090	2.197 ± 0.205	ns
Tissue lipid concentration (mg cm^−2^)	0.227 ± 0.008	0.215 ± 0.011	0.281 ± 0.013	Pulse > Control, Press
Tissue protein concentration (mg cm^−2^)	1.766 ± 0.072	1.736 ± 0.132	1.495 ± 0.072	ns
Tissue N:P ratio	13.981 ± 0.271	13.534 ± 0.481	12.969 ± 0.162	ns
Tissue organic C content (% weight)	3.408 ± 0.059	4.150 ± 0.032	5.278 ± 0.059	Pulse > Press > Control

## Discussion

**Figure 3:**
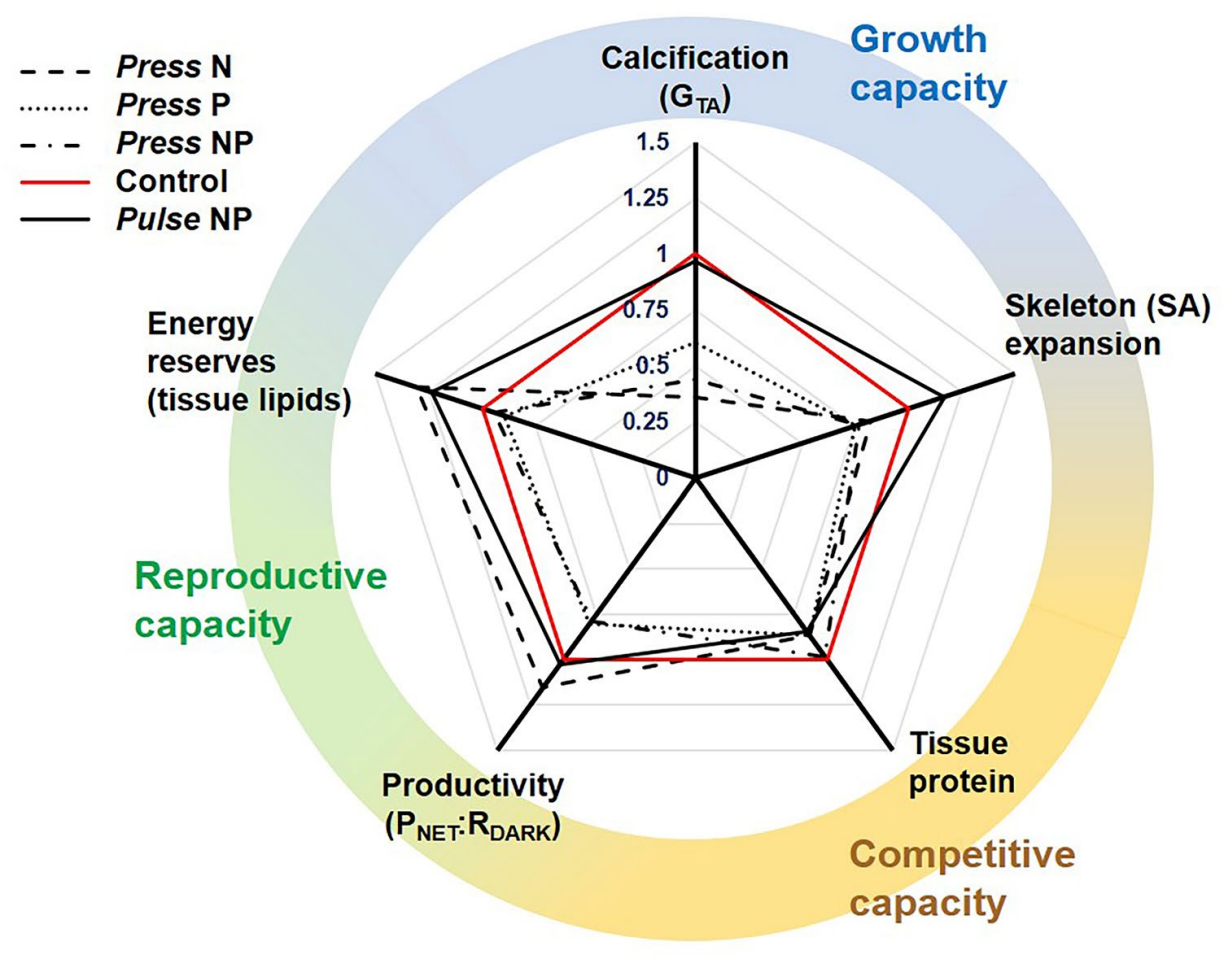
Conceptual summary depicting changes or trade-offs in survival strategy of *Acropora intermedia* under different treatments of elevated ammonium (N) and/or phosphate (P), as permanent (*press*) or temporary (*pulse*) elevations. To create this plot, the average performance for each physiological parameter (calcification, skeleton expansion, tissue protein, productivity, or stored energy reserves) in a treatment is calculated and represented as a fraction of the respective parameter for the control treatment, which is always fixed at 1 (red line). The three main strategies of long-term colony viability are represented on the colored circle: growth (blue), competition (orange), and reproduction (green). Here, growth is defined as the combined capacity to amass skeleton and energy reserves, competition as the capacity to occupy or consolidate physical space on the reef, and reproduction as the capacity to produce offspring. Calcification was calculated as the 24 h-average of day and nighttime calcification based on a 12–12 h day–night cycle.

This study reveals that perturbations to local nutrient levels can significantly affect key aspects of the physiology of *Acropora intermedia*, a common reef-building coral on the Great Barrier Reef. Inorganic nutrient levels are increasing in coastal waters where many coral reefs grow^63^. Understanding how local pressures influence corals and their symbionts is timely and important, particularly in the context of a rapidly changing global environment. Our results demonstrate (1) how multiple nutrients do not interact to produce synergistic or antagonistic outcomes, and (2) how *A. intermedia* benefits from episodic pulses of concentrated nutrients. Together, these findings highlight the importance of considering nutrient form, stoichiometry and origin on coral reefs. Overall, under permanently elevated ammonium and phosphate *A. intermedia* allocated resources towards tissue growth at the expense of skeletogenesis (Fig. 3).

### Individual and combined effects of press elevated ammonium and phosphate

*Press* elevated levels of ammonium or phosphate induced contrasting physiological responses in *A. intermedia*. Elevated levels of ammonium led to a substantial decrease in CaCO_3_ accretion rates. Primary productivity and tissue lipid concentration were unaffected. By contrast, elevated phosphate did not affect net CaCO_3_ accretion rates, but it reduced skeletal surface area expansion, a measure of the coral’s potential to expand and occupy new territory. Additionally, elevated phosphate reduced primary productivity rates and tissue lipid concentrations per surface area. We found that ammonium and phosphate rarely produced interactive effects when applied together, and most physiological parameters assessed in this study were individually influenced by one of the two nutrients.

Calcification rates (G_TA_) of *A. intermedia* under daytime conditions decreased after 7 weeks of exposure to *press* elevated ammonium concentrations of 14 µmol L^−1^. This is consistent with previous studies which found reduced calcification under high concentrations (10–109 µmol L^−1^) of ammonium^20, 29, 32^, and nitrate^25, 26, 64^. Other studies found increased skeletal growth for several coral species reared under mild concentrations of 2 and 5 µmol L^−1^ of ammonium and nitrate respectively^65^, or heterogenous effects under 11.3–36.2 µmol L^−1^ ammonium^5^. Similarly, studies on coral calcification (mainly linear expansion rates and buoyant weight measurements) under elevated phosphate have reported mixed effects for different coral species, describing both enhancement^5, 66, 67^ and reduction^5, 20, 29, 32^ under phosphate concentrations ranging from 0.5 to 13 µmol L^−1^. Skeletal surface area increase in the present study was reduced under elevated phosphate concentrations (10 µmol L^−1^), despite no observed changes in net CaCO_3_ accretion. This indicates a shift towards higher skeletal density and lower skeletal expansion, thus reducing the competitive ability of *A. intermedia* to occupy new territory, while gaining improved tolerance to physical damage and fragmentation^68^. Such shifts are notably beneficial in upwelling areas characterized by high nutrients but also intensified wave energy^69^.

Despite the highest P_NET_:R_DARK_ ratios measured in the press N treatment, productivity was not significantly stimulated under elevated N. Previous studies report up to 5 times higher symbiont densities, and 150% increase in primary productivity under elevated ammonium concentrations^20, 24, 47^, identifying nitrogen availability as a limiting factor for productivity alongside bicarbonate^21, 70^. Possible benefits of increased nitrogen availability could have been balanced by increased symbiont competition for other resources^27, 30^. Similarly, productivity decreased under press phosphate addition, possibly due to higher symbiont densities and increased self-shading^24, 71^. Excess photosynthetic carbon could have been stored as somatic lipid reserves^58^, explaining elevated tissue lipid but not protein content under increased ammonium. Alternatively, excess autotrophic carbon could have been allocated towards other processes not assessed here such as increased host mucus production^72^ or symbiont population^16, 47^. There is growing evidence that symbiont growth reduces carbon translocation to the host, and thereby influences host processes such as skeletogenesis^16, 28–30^. Strong competition for DIC between a larger symbiont population (for photosynthesis) and the host (for calcification) could explain reduced coral skeletal expansion and CaCO_3_ accretion rates under *press* elevated nutrients.

The accumulation of organic carbon in the holobiont tissue was unchanged by the addition of ammonium or phosphate individually but increased when the two were added as a combined treatment. This suggests co-limitation of these two nutrients to biomass production in the holobiont^23, 66, 73^ under ambient conditions on Heron Island. Tissue TOC and lipid content was further increased under the *pulse* NP treatment, where the highest tissue TOC and lipid content were recorded. Interestingly, holobiont N:P ratios were unaffected by the addition of ammonium and/or phosphate, neither as *press* nor *pulse* treatments, despite the imbalanced N:P ratio of the seawater (Table S1). This suggests no differential increase in the assimilation of these nutrients. Muller-Parker et al. (1994a) reported N enrichment in Symbiodiniaceae but not in the host after 8 weeks exposure to 20–50 µmol L^−1^ ammonium, further supporting that nutrients are primarily utilized by Symbiodiniaceae, and that nutrient enrichment benefits the symbiont rather than the host. The uptake of ammonium or phosphate and subsequent shift in holobiont N:P ratio may have been diluted here, given that N and P are predominantly assimilated by Symbiodiniaceae and the microbial community of the holobiont^74, 75^, which occupy a relatively small fraction of the holobiont mass^22^, and have limited nutrient storage capabilities^76^.

### Benefits of episodic nutrient addition

Concentrations of inorganic N and P in seawater overlying coral reefs are generally low, and nutrient enrichment has often been negatively associated with coral growth^5, 26, 48^. Interestingly, fish-derived nutrients are known to improve coral physiology^37, 38, 43, 44^, yet the underlying stoichiometry and physiological mechanisms are not yet investigated^48^. Shantz and Burkepile (2014) concluded that anthropogenic nutrient enrichment tended to negatively influence overall coral performance while biologically mediated nutrients generally had positive effects, and hypothesized that the pulsed nature of natural nutrient enrichment could be responsible. Corals under unperturbed conditions are naturally accustomed to high concentration nutrient pulses^37, 38, 77^, but the daily migratory and feeding behaviour of reef organisms mean that these nutrients fluctuate considerably^37, 38^, simulated by our *pulse* treatment. We provide further evidence that short, strong nutrient pulses benefit *A. intermedia* compared to continuously elevated nutrients, and for some parameters even compared to unmanipulated control conditions. Rates of day and nighttime G_TA_, P_NET_:R_DARK_, fragment surface area, and holobiont tissue TOC and lipid content were all distinctly higher in the *pulse* NP treatment compared to the *press* NP treatment, while lipid concentration, holobiont TOC content, night-time GT_A_ and fragment surface area even increased compared to the control treatment.

Pulsed addition of anthropogenic nutrients has previously been found to positively influence coral communities^5^ as well as other aquatic ecosystems such as seagrass beds and macroalgae communities^78, 79^. Our results indicate that the brevity of the pulse possibly plays a key role in the coral’s ability to manage high nutrient concentrations. Non-linearity between continuous nutrient exposure and coral calcification has previously been shown, suggesting that low nutrient doses (i.e. nutrient *pulse* in our study) enhanced calcification while high doses (i.e. nutrient *press*) reduced calcification^80^. Rapid uptake and assimilation of phosphate and ammonium by Symbiodiniaceae^81^, oxidation of ammonium to nitrite and nitrate by the coral microbial associates^74, 75^, or direct host ammonium assimilation through the GS/GDH pathway^82^ all potentially mitigated nutrient impact during short pulses, allowing corals to profit. Overall, fragments that received nutrient pulses had improved growth (both as CaCO_3_ accretion and skeleton expansion) and energy reserves, without trade-offs in other fields such as productivity. On the other hand, corals under *press* elevated N and/or P were able to consolidate their productivity, tissue protein and lipid stores, but at the expense of much of their skeletal growth (Fig. 3). Such trade-offs could result in immediate changes to the competitive balance within reef communities and compromise long-term coral viability^34, 83, 84^.

## Conclusions

Inorganic nutrients remain important factors in influencing physiology in coral-dinoflagellate symbioses. The present study provides insights into the stoichiometry of two of the most important inorganic nutrients, ammonium and phosphate, and their individual and combined influence on parameters of skeletogenesis, photobiology and tissue properties in *A. intermedia*. At the same time, ammonium and phosphate concentrations that would normally compromise coral health if maintained permanently, are shown to benefit corals when administered in shorter pulses. Together, these results underline the complex and context-specific relationship between coral holobiont physiology and elevated nutrients.

## Supplementary Information

Supplementary Information.
