# Supplementary material for: Asymmetric physiological response of a reef-building coral to pulsed versus continuous addition of inorganic nutrients
Source: Sci Rep. 2021 Jun 23;11:13165. doi: 10.1038/s41598-021-92276-y (PMC8222273; doi:10.1038/s41598-021-92276-y)
Supplement: Supplementary file 1 — Supplementary Information. [file 41598_2021_92276_MOESM1_ESM.docx]

**Asymmetric physiological response of a reef-building coral to pulsed versus continuous addition of inorganic nutrients**

Rene M. van der Zande*, Yannick R. Mulders, Dorothea Bender-Champ, Ove Hoegh-Guldberg, Sophie Dove.

* Corresponding author:

Rene M van der Zande. rene.vanderzande@uq.net.au

**Table of contents:**

Supplementary methods…………………………………………………………………………..……..2

Total alkalinity measurement corrections………………………………………………………2

Tissue composition analysis………………………………………………………………….…3

Supplementary Figures…………………………………………………………………………………..4

Figure S1. Daily ammonium and phosphate in the treatment aquaria………………………4

Figure S2. Dynamics of aquarium N and P before, during and after nutrient pulse………...5

Supplementary Tables……………………………………………………………………………………6

Table S1. Ammonium and phosphate conditions in the treatment aquaria…………………6

Table S2. Ammonium and phosphate concentrations and uptake rates ….………………..7

Table S3. Results of all statistical analyses………………..…………………………………..8

References………………………………………………………………………………………….……12

**Supplementary methods**

*Total alkalinity measurement corrections*

Initial seawater total alkalinity (TA) was determined from a triplicate seawater sample collected at the start of the incubation from the bulk incubation water, while end-point seawater TA was determined from seawater samples collected from each incubation chamber. TA was determined by Gran titration after ^1^ (Mettler-Toledo T50 titrator, Mettler-Toledo, Greifensee, Switzerland). Additional water samples were collected from the bulk incubation water (before) and incubation chambers (after) to measure ammonium and phosphate uptake/release rates by the coral during the incubation as these can affect the alkalinity precision (Table S2). Ammonium and phosphate concentrations were determined according to ^2^ (pp. 14-17 and pp. 22-25 for ammonium and phosphate respectively). The changes in TA attributable to calcification (ΔTA_CaCO3_) were calculated by correcting the total TA change (ΔTA_total_) for changes in ammonium (ΔTA_NH4_) and phosphate (ΔTA_PO4_) concentration during the incubation period (Eq. 1) according to ^3^. Total CaCO_3_ accretion rates (G_TA_) were then calculated using Eq. 2.

$\Delta{{TA}}_{CaCO3}= \Delta{TA}_{total}- \Delta{TA}_{NH4}- \Delta{TA}_{PO4}$ Eq. 1

$G_{TA} \left( \mu mol {CaCO}_{3} {cm}^{-2} h^{-1} \right)= \left( \frac{\Delta{TA}_{CaCO3} \left( \mu mol \right)}{2 \cdot{SA}_{end} \left( {cm}^{2} \right)\cdot time \left( h \right)} \right) \cdot Vol \left( L \right)$ Eq. 2

*Tissue composition analyses*

After the incubations the coral fragments were frozen for tissue analysis (n = 12 per treatment). Tissue was removed from the skeleton using a simple airbrush with 10 ml, 0.45 µm filtered seawater. Half the tissue mixture (5 ml) was frozen for N:P ratio analysis (host and symbiont combined). Of the remaining 5 ml tissue mixture, half (2.5 ml) was frozen at -20 °C for lipid analysis and the other half was centrifuged down for mass separation at 4500 RPM for 5 min. Two ml of the supernatant was collected for host protein analysis and the rest of the supernatant was discarded. Water-soluble host protein was determined by differential absorbance at 235 and 280 nm through spectrometry (Spectra Max 2, Molecular Devices, Sunnyvale, California) after ^4^.

Lipids were quantified according to a modified protocol from ^5^. The 2.5 ml frozen lipid sample was freeze dried (ScanVac CoolSafe, LaboGene, Lillerød, Denmark), and afterwards suspended in 5 ml chloroform/methanol (2:1) solution, vortexed and left overnight in the dark at 4 °C for lipid extraction. The liquid fraction of the suspension was removed into a new falcon tube, and the remaining pellet was rinsed with 2 ml of the chloroform/methanol solution. After one hour at 4 °C in the dark, this 2 ml suspension was added to the initial 5 ml liquid fraction sample. After this step 1 ml of 0.1 mol L^-1^ KCl solution was added to the sample, vortexed again and left overnight in the dark at 4 °C to let the organic and aqueous phases separate. The aqueous phase was pipetted off, and the organic phase was rinsed with 5 ml of methanol/Milli-Q (1:1) and stored in the dark at 4 °C. After renewed separation of the phases, the aqueous phase was discarded, and the rinsing process was repeated two more times. After the final rinse, the organic solution was poured into pre-weighed aluminium trays, dried, and reweighed for lipid quantification.

The frozen 5 ml sample set aside for N:P analysis was pooled by tank (n = 4 per treatment) and freeze dried (ScanVac CoolSafe, LaboGene, Lillerød, Denmark). The resulting dry material was weighed into three separate portions, for total nitrogen (TN), total phosphorus (TP) and total organic carbon (TOC) analyses. TN was analysed by combustion analysis (LECO TruSpec analyser, Michigan, USA), TP was analysed by acid digestion and inductively coupled plasma optical emission spectrometry (ICP-OES) analysis, and TOC was analysed by combustion (LECO TruSpec analyser, Michigan, USA) on acidified samples to remove carbonates. Results from the TN and TP analysis were combined for N:P ratio determination.


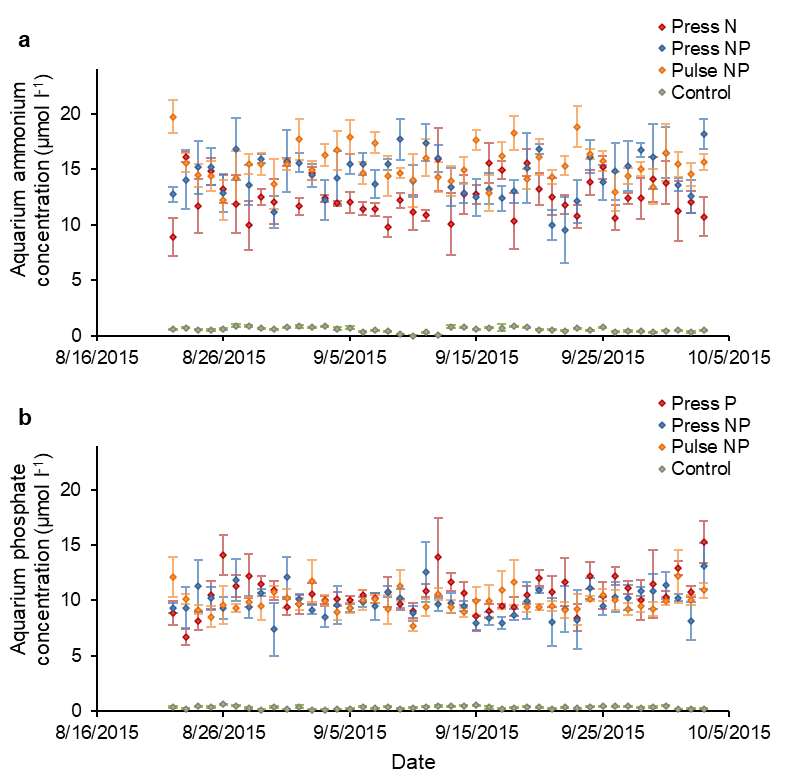


**Figure S1.** Average ammonium (**a**) and phosphate (**b**) concentrations (mean ± SE) in the experimental aquaria (n = 4 per treatment) throughout the experimental period. Samples from the *Pulse* NP treatment were taken during the elevated nutrient phase.


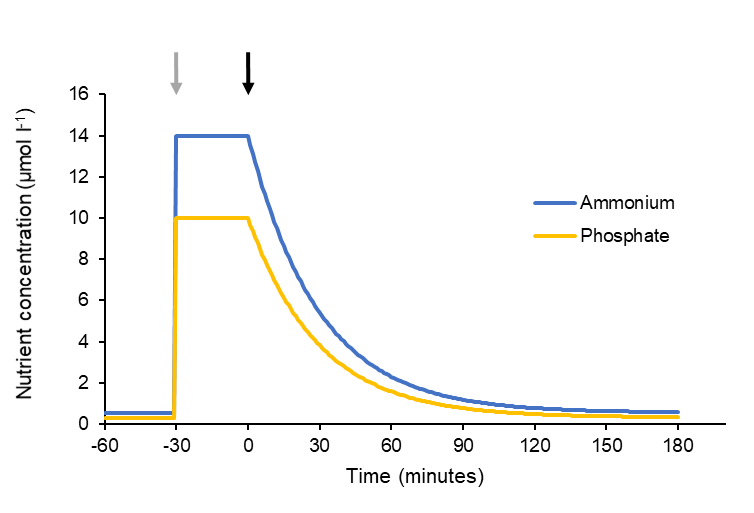


**Figure S2.** Theoretical dynamics of ammonium (blue) and phosphate (yellow) concentration inside the *pulse* aquaria (30 L volume) before, during and after the *pulse* delivery. In the *pulse* treatment, water flow in the aquaria was interrupted (grey arrow) for 30 minutes and ammonium and phosphate in the aquaria were for that period increased to 14 and 10 µmol L^-1^ respectively based on treatment objectives. After the 30-minute period, water flow was resumed (black arrow) and the nutrient concentration steadily declined to background concentrations. In this calculation of nutrient decline, ammonium and phosphate concentrations of the flow-through water are assumed at 0.55 and 0.32 µmol L^-1^ respectively, based on control treatment averages for Heron Island (Table S1). Water flow is resumed at 1 L min^-1^, and biological uptake of nutrients by the corals in the aquaria is not accounted for. At 180 minutes after restart the conditions inside the aquaria again approximate background nutrient conditions.

**Table S1.** Comparison of the average ammonium and phosphate concentrations and doses (mean ± SE) that were realized between the different treatments across the 7-week treatment period. Nutrients in the *press* N, P and NP treatments were maintained permanently elevated at an intended concentration of 14 and 10 µmol L^-1^ ammonium and phosphate respectively. The *pulse* NP treatment consisted of nutrient pulses of the same concentration as the *press* treatments added twice a day and maintained for 30 minutes. Nutrient concentrations were measured before and after the pulse was administered. The control treatment received untreated seawater with ambient nutrient concentrations.

|  | |  | Average concentrations in tanks (mean ± SE) | |  |
| --- | --- | --- | --- | --- | --- |
|  | |  | Ammonium | Phosphate | Average seawater N:P ratio |
| Treatment | |  | (µmol L^-1^) | (µmol L^-1^) | (NH_4_ : PO_4_) |
|  | |  |  |  |  |
| Control | |  | 0.55 ± 0.02 | 0.32 ± 0.01 | 1.73 |
| *Press* N | |  | 14.41 ± 0.25 | 0.32 ± 0.01 | 45.09 |
| *Press* P | |  | 0.55 ± 0.02 | 10.70 ± 0.24 | 0.05 |
| *Press* NP | |  | 12.36 ± 0.37 | 9.70 ± 0.21 | 1.27 |
| *Pulse* NP | Before |  | 0.55 ± 0.02 | 0.32 ± 0.01 | 1.73 |
|  | After |  | 15.39 ± 0.63 | 9.91 ± 0.32 | 1.55 |

**Table S2.** Average concentrations (A) and uptake rates (B) of ammonium and phosphate during the calcification measurements under light and dark conditions (mean ± SE). Uptake rates were measured during the light and dark calcification incubations at the end of the 7-week exposure to the elevated nutrient treatments and standardized to fragment surface area. Absolute ammonium and phosphate uptake per incubation chamber was used to correct the alkalinity values used for the calcification (G_TA_) measurements.

| Treatment | (**A**) Concentrations (µmol L^-1^) | | | |
| --- | --- | --- | --- | --- |
|  | NH_4_ | | PO_4_ | |
|  | Light | Dark | Light | Dark |
| Control | 0.36 ± 0.04 | 0.53 ± 0.06 | 0.33 ± 0.02 | 0.30 ±0.04 |
| *Press* N | 10.78 ± 0.29 | 15.49 ± 1.13 | 0.21 ± 0.05 | 0.42 ± 0.03 |
| *Press* P | 0.68 ± 0.06 | 0.68 ± 0.05 | 10.38 ± 0.45 | 14.11 ± 1.44 |
| *Press* NP | 16.82 ± 0.98 | 17.77 ± 1.17 | 12.21 ± 1.16 | 12.24 ± 1.03 |
| *Pulse* NP | 0.52 ± 0.05 | 0.64 ± 0.04 | 0.30 ± 0.05 | 0.25 ± 0.02 |
|  |  |  |  |  |
|  | (**B**) Uptake (nmol cm^-2^ h^-1^) | | | |
| Treatment | NH_4_ | | PO_4_ | |
|  | Light | Dark | Light | Dark |
| Control | 3.54 ± 1.14 | -0.79 ± 0.82 | 3.05 ± 0.87 | 1.35 ± 0.98 |
| *Press* N | 109.20 ± 13.49 | 17.68 ± 6.97 | 2.03 ± 0.88 | 1.02 ± 0.66 |
| *Press* P | -13.90 ± 1.94 | -14.30 ± 2.07 | -0.10 ± 0.91 | 1.42 ± 1.39 |
| *Press* NP | 65.04 ± 20.46 | 102.41 ± 17.05 | 4.54 ± 2.20 | -6.63 ± 9.48 |
| *Pulse* NP | -4.09 ± 1.70 | -5.50 ± 2.29 | -1.25 ± 1.50 | -1.58 ± 0.92 |

**Table S3.** Results of all statistical analyses of the physiological parameters discussed in the main article. All variables were analysed in a factorial analysis to assess the individual and interactive effects of elevated ammonium and phosphate (A), and in one-way ANOVA design to assess the difference between *press* and *pulse* nutrient addition (B). In the factorial comparison, the variables net photosynthesis (P_NET_), dark respiration (R_DARK_), P_NET_:R_DARK_ ratio, end-of-experiment fragment surface area, tissue lipid content and tissue protein content were analysed in a nested two-factorial design (factors: N and P, levels: ambient and elevated), with the treatment tank nested in the interaction of the factors. End-of-experiment calcification rates (G_TA_) was analysed for tank effects in a preliminary analysis, and then using a three-way repeated measures design (factors N, P and TIME; levels ambient and elevated for factors N and P, and levels day and night for factor TIME) to compare light and dark G_TA_ rates. Tissue N:P ratios and organic carbon (TOC) content were analysed at tank level in a two-way factorial design (levels ambient and elevated for factors N and P). In the press - pulse comparison, all variables except for G_TA_, tissue N:P ratio and tissue TOC content were analysed in a nested one-way ANOVA design, with tank nested in the treatments. G_TA_ was analysed in a repeated measures one-way ANOVA. Tissue N:P ratio and tissue TOC content were analysed at tank level in a one-way ANOVA. Red typeface indicates significance, and the * sign indicates datasets that were analysed at α = 0.01 due to violationsof heteroscedascity.

|  |  |  |  |  |  |  |  |  |  |  |
| --- | --- | --- | --- | --- | --- | --- | --- | --- | --- | --- |
|  |  | **Variable** | **Analysis** |  | **Source of** |  | **DF** | **MS** | **F** | **p** |
|  |  |  |  |  | **variation** |  |  |  |  |  |
|  |  |  |  |  |  |  |  |  |  |  |
|  |  |  |  |  |  |  |  |  |  |  |
| **A. Factorial analysis** | | |  |  |  |  |  |  |  |  |
|  |  |  |  |  |  |  |  |  |  |  |
|  | **1** | **End-of-experiment rate** | 3-way ANOVA |  | N |  | 1 | 0.043240 | 9.0358 | 0.004362 |
|  |  | **of calcification (G_TA_)** | (repeated measures) |  | P |  | 1 | 0.006892 | 1.4402 | 0.236530 |
|  |  | **(µmol CaCO_3_ cm^-2^ h^-1^) *** |  |  | N × P |  | 1 | 0.015268 | 3.1906 | 0.080954 |
|  |  |  |  |  | Error |  | 44 | 0.004785 |  |  |
|  |  |  |  |  | TIME |  | 1 | 0.398225 | 143.4975 | 0.000000 |
|  |  |  |  |  | TIME × N |  | 1 | 0.030482 | 10.9839 | 0.001846 |
|  |  |  |  |  | TIME × P |  | 1 | 0.001782 | 0.6420 | 0.427286 |
|  |  |  |  |  | TIME × N × P | | 1 | 0.001394 | 0.5022 | 0.482274 |
|  |  |  |  |  | Error |  | 44 | 0.002775 |  |  |
|  |  |  |  |  |  |  |  |  |  |  |
|  | **1a** | **G_TA_ (light conditions)** | Preliminary test for |  | N |  | 1 | 0.073165 | 10.8509 | 0.002417 |
|  |  | **(µmol CaCO_3_ cm^-2^ h^-1^)** | tank effects |  | P |  | 1 | 0.007841 | 1.1629 | 0.288933 |
|  |  |  |  |  | N × P |  | 1 | 0.003718 | 0.5514 | 0.463156 |
|  |  |  |  |  | Tank (N × P) | | 12 | 0.006988 | 1.0364 | 0.441600 |
|  |  |  |  |  | Error |  | 32 | 0.006743 |  |  |
|  |  |  |  |  |  |  |  |  |  |  |
|  | **1b** | **G_TA_ (dark conditions)** | Preliminary test for |  | N |  | 1 | 0.000556 | 0.78603 | 0.381919 |
|  |  | **(µmol CaCO_3_ cm^-2^ h^-1^)** | tank effects |  | P |  | 1 | 0.000833 | 1.17676 | 0.286122 |
|  |  |  |  |  | N × P |  | 1 | 0.012944 | 18.29413 | 0.000160 |
|  |  |  |  |  | Tank (N × P) | | 12 | 0.000867 | 1.22473 | 0.309447 |
|  |  |  |  |  | Error |  | 32 | 0.000708 |  |  |
|  |  |  |  |  |  |  |  |  |  |  |
|  | **2** | **P_NET_:R_DARK_ ratio** | Nested 2-way ANOVA |  | N |  | 1 | 0.2867 | 1.556 | 0.221263 |
|  |  |  |  |  | P |  | 1 | 4.4361 | 24.083 | 0.000026 |
|  |  |  |  |  | N × P |  | 1 | 0.3911 | 2.123 | 0.154804 |
|  |  |  |  |  | Tank (N × P) | | 12 | 0.0956 | 0.519 | 0.886629 |
|  |  |  |  |  | Error |  | 32 | 0.1842 |  |  |
|  |  |  |  |  |  |  |  |  |  |  |
|  | **3** | **Net photosynthesis** | Nested 2-way ANOVA |  | N |  | 1 | 0.00398 | 0.0437 | 0.835796 |
|  |  | **rate (P_NET_)** |  |  | P |  | 1 | 0.38728 | 4.2443 | 0.047593 |
|  |  | **(µmol O_2_ cm^-2^ h^-1^) *** |  |  | N × P |  | 1 | 0.00772 | 0.0846 | 0.773077 |
|  |  |  |  |  | Tank (N × P) | | 12 | 0.06671 | 0.7311 | 0.711255 |
|  |  |  |  |  | Error |  | 32 | 0.09125 |  |  |
|  |  |  |  |  |  |  |  |  |  |  |
|  | **4** | **Dark respiration** | Nested 2-way ANOVA |  | N |  | 1 | 0.021912 | 1.8843 | 0.179387 |
|  |  | **Rate (R_DARK_)** |  |  | P |  | 1 | 0.000437 | 0.0376 | 0.847489 |
|  |  | **(µmol O_2_ cm^-2^ h^-1^)** |  |  | N × P |  | 1 | 0.001862 | 0.1602 | 0.691676 |
|  |  |  |  |  | Tank (N × P) | | 12 | 0.018368 | 1.5796 | 0.147769 |
|  |  |  |  |  | Error |  | 32 | 0.011628 |  |  |
|  |  |  |  |  |  |  |  |  |  |  |
|  | **5** | **Fragment surface area** | Nested 2-way ANOVA |  | N |  | 1 | 17.15 | 2.025 | 0.161210 |
|  |  | **(cm^2^)** |  |  | P |  | 1 | 56.40 | 6.660 | 0.012975 |
|  |  |  |  |  | N × P |  | 1 | 21.90 | 2.585 | 0.114411 |
|  |  |  |  |  | Tank (N × P) | | 12 | 7.73 | 0.912 | 0.541971 |
|  |  |  |  |  | Error |  | 48 | 8.47 |  |  |
|  |  |  |  |  |  |  |  |  |  |  |
|  | **6** | **Tissue lipid content** | Nested 2-way ANOVA |  | N |  | 1 | 0.020 | 11.59 | 0.001805 |
|  |  | **(mg cm^-2^) *** |  |  | P |  | 1 | 0.034 | 19.77 | 0.000098 |
|  |  |  |  |  | N × P |  | 1 | 0.010 | 6 | 0.019969 |
|  |  |  |  |  | Tank (N × P) | | 12 | 0.001 | 0.64 | 0.796371 |
|  |  |  |  |  | Error |  | 32 | 0.001721 |  |  |
|  |  |  |  |  |  |  |  |  |  |  |
|  | **7** | **Tissue protein content** | Nested 2-way ANOVA |  | N |  | 1 | 0.0024 | 0.0162 | 0.899605 |
|  |  | **(mg cm^-2^)** |  |  | P |  | 1 | 0.0028 | 0.0187 | 0.892051 |
|  |  |  |  |  | N × P |  | 1 | 0.5770 | 3.8182 | 0.059486 |
|  |  |  |  |  | Tank (N × P) | | 12 | 0.1244 | 0.8232 | 0.626388 |
|  |  |  |  |  | Error |  | 32 | 0.1511 |  |  |
|  |  |  |  |  |  |  |  |  |  |  |
|  | **8** | **Holobiont N:P ratio** | 2-way ANOVA |  | N |  | 1 | 2.138 | 4.075 | 0.066440 |
|  |  |  |  |  | P |  | 1 | 0.332 | 0.632 | 0.441956 |
|  |  |  |  |  | N × P |  | 1 | 0.051 | 0.097 | 0.760984 |
|  |  |  |  |  | Error |  | 12 | 0.525 |  |  |
|  |  |  |  |  |  |  |  |  |  |  |
|  | **9** | **Holobiont TOC content** | 2-way ANOVA |  | N |  | 1 | 0.7526 | 126.44 | 0.000000 |
|  |  | **(% of weight)** |  |  | P |  | 1 | 0.3813 | 64.06 | 0.000004 |
|  |  |  |  |  | N × P |  | 1 | 0.1580 | 26.55 | 0.000240 |
|  |  |  |  |  | Error |  | 12 | 0.0060 |  |  |
|  |  |  |  |  |  |  |  |  |  |  |
|  |  |  |  |  |  |  |  |  |  |  |
| **B. Press - Pulse comparison** | | |  |  |  |  |  |  |  |  |
|  |  |  |  |  |  |  |  |  |  |  |
|  | **10** | **End-of-experiment rate of** | *Press - Pulse* |  | Treatment |  | 2 | 0.008883 | 6.0568 | 0.021556 |
|  |  | **calcification (G_TA_)** | comparison (repeated |  | Error |  | 9 | 0.001467 |  |  |
|  |  | **(µmol CaCO_3_ cm^-2^ h^-1^)** | measures ANOVA) |  | Time |  | 1 | 0.076751 | 51.0599 | 0.000054 |
|  |  |  |  |  | Time × Treatment | | 2 | 0.004310 | 2.8671 | 0.108797 |
|  |  |  |  |  | Error |  | 9 | 0.001503 |  |  |
|  |  |  |  |  |  |  |  |  |  |  |
|  | **11** | **Net photosynthesis** | *Press - Pulse* |  | (Tank) Treatment | | 9 | 0.06530 | 1.0995 | 0.399585 |
|  |  | **rate (P_NET_)** | comparison (nested |  | Treatment |  | 2 | 0.14928 | 2.5134 | 0.102079 |
|  |  | **(µmol O_2_ cm^-2^ h^-1^)** | one-way ANOVA) |  | Error |  | 24 | 0.05939 |  |  |
|  |  |  |  |  |  |  |  |  |  |  |
|  | **12** | **Dark respiration** | *Press - Pulse* |  | (Tank) Treatment | | 9 | 0.019240 | 1.0807 | 0.411589 |
|  |  | **rate (R_DARK_)** | comparison (nested |  | Treatment |  | 2 | 0.016708 | 0.9385 | 0.405112 |
|  |  | **(µmol O_2_ cm^-2^ h^-1^)** | one-way ANOVA) |  | Error |  | 24 | 0.017803 |  |  |
|  |  |  |  |  |  |  |  |  |  |  |
|  | **13** | **P_NET_:R_DARK_ ratio** | *Press - Pulse* |  | (Tank) Treatment | | 9 | 0.0840 | 0.2750 | 0.975500 |
|  |  |  | comparison (nested |  | Treatment |  | 2 | 0.9390 | 3.0742 | 0.064771 |
|  |  |  | one-way ANOVA) |  | Error |  | 24 | 0.3055 |  |  |
|  |  |  |  |  |  |  |  |  |  |  |
|  | **14** | **Fragment surface area** | *Press - Pulse* |  | (Tank) Treatment | | 9 | 14.27 | 1.494 | 0.187494 |
|  |  | **(cm^2^)** | comparison (nested |  | Treatment |  | 2 | 102.43 | 10.722 | 0.000222 |
|  |  |  | one-way ANOVA) |  | Error |  | 36 | 9.55 |  |  |
|  |  |  |  |  |  |  |  |  |  |  |
|  | **15** | **Tissue lipid content** | *Press - Pulse* |  | (Tank) Treatment | | 9 | 0.000565 | 0.340 | 0.952038 |
|  |  | **(mg cm^-2^)** | comparison (nested |  | Treatment |  | 2 | 0.015039 | 9.048 | 0.001179 |
|  |  |  | one-way ANOVA) |  | Error |  | 24 | 0.001662 |  |  |
|  |  |  |  |  |  |  |  |  |  |  |
|  | **16** | **Tissue protein content** | *Press - Pulse* |  | (Tank) Treatment | | 9 | 0.12638 | 0.7432 | 0.666801 |
|  |  | **(mg cm^-2^)** | comparison (nested |  | Treatment |  | 2 | 0.26597 | 1.5641 | 0.229870 |
|  |  |  | one-way ANOVA) |  | Error |  | 24 | 0.17004 |  |  |
|  |  |  |  |  |  |  |  |  |  |  |
|  | **17** | **Holobiont N:P ratio** | *Press - Pulse* comparison |  | Treatment |  | 2 | 1.030 | 2.331 | 0.152840 |
|  |  |  | (one-way ANOVA) |  | Error |  | 9 | 0.442 |  |  |
|  |  |  |  |  |  |  |  |  |  |  |
|  |  |  |  |  |  |  |  |  |  |  |
|  | **18** | **Holobiont TOC content** | *Press - Pulse* comparison |  | Treatment |  | 2 | 3.5463 | 333.33 | 0.000000 |
|  |  | **(% of weight)** | (one-way ANOVA) |  | Error |  | 9 | 0.0106 |  |  |
|  |  |  |  |  |  |  |  |  |  |  |
|  |  |  |  |  |  |  |  |  |  |  |

**References**

1. Dickson, A. G., Afghan, J. D. & Anderson, G. C. Reference materials for oceanic CO2 analysis: a method for the certification of total alkalinity. *Mar. Chem.* **80**, 185–197 (2003).

2. Parsons, T. R., Maita, Y. & Lalli, C. M. *A manual of chemical and biological methods for seawater analysis*. (Pergamon Press, 1984).

3. Maier, C., Watremez, P., Taviani, M., Weinbauer, M. G. & Gattuso, J. P. Calcification rates and the effect of ocean acidification on Mediterranean cold-water corals. *Proc. R. Soc. B Biol. Sci.* **279**, 1716–1723 (2012).

4. Whitaker, J. R. & Granum, P. E. An absolute method for protein determination based on difference in absorbance at 235 and 280 nm. *Anal. Biochem.* **109**, 156–159 (1980).

5. Dunn, S. R., Thomas, M. C., Nette, G. W., Dove, S. G. & Blackburn, S. A lipidomic approach to understanding free fatty acid lipogenesis derived from dissolved inorganic carbon within Cnidarian-Dinoflagellate symbiosis. *PLoS One* **7**, e46801 (2012).
